# Lightweight CNN-Based Visual Perception Method for Assessing Local Environment Complexity of Unmanned Surface Vehicle

**DOI:** 10.3390/s25030980

**Published:** 2025-02-06

**Authors:** Tulin Li, Xiufeng Zhang, Yingbo Huang, Chunxi Yang

**Affiliations:** Yunnan Key Laboratory of Intelligent Control and Application, Faculty of Mechanical and Electrical Engineering, Kunming University of Science and Technology, Kunming 650500, China; litulin@stu.kust.edu.cn (T.L.); 20220056@kust.edu.cn (X.Z.); Yingbo_Huang@kust.edu.cn (Y.H.)

**Keywords:** unmanned surface vehicle (USV), convolutional neural network (CNN), residual learning, environmental perception, lightweight network, multi-feature fusion, complexity identification and classification

## Abstract

Addressing the problem of inadequate environmental detection in the process of optimizing search for unmanned surface vehicles (USVs) by a heuristic algorithm, this paper proposes a comprehensive visual perception method that combines a lightweight convolutional neural network (CNN) with the USV’s real-time heading angle. This method employs a multi-feature input CNN with residual learning blocks, which takes both the current local environmental images and heading angle features as inputs to identify the complexity of the local environment with higher accuracy and a smaller load size. Meanwhile, human expertise is incorporated to classify labels through a majority voting system, thereby making the model’s perceptual classification more intuitive and allowing it to possess a human-like comprehensive perception ability compared to systems with classification methods with several parameters. Subsequently, this identification result can be used as feedback for the heuristic algorithm to optimize and plan the USV’s path. The simulation results indicate that the developed model achieves an 80% reduction in model size while maintaining an accuracy exceeding 90%. The proposed method significantly improves the environment recognition capability of the heuristic algorithm, enhances optimization search efficiency, and increases the overall performance of path planning by approximately 21%.

## 1. Introduction

With the rapid advancement and widespread application of unmanned surface vehicle (USVs), the enhancement of their autonomy emerges as a pivotal trend in technological evolution [1]. Consequently, the development and deployment of USVs tailored to diverse practical application scenarios have increasingly garnered heightened attention [2,3]. The issue of path planning holds a crucial position in safeguarding the navigational safety of USVs, influencing multiple aspects including payload capacity, endurance, and maneuverability. In this context, the precision and dependability of environmental perception constitute a fundamental prerequisite for assessing the rationality and efficacy of the planning process.

Environmental perception typically involves the creation of a simulated map that mirrors the real environment, from which environmental information pertinent to the USV is extracted. This information subsequently guides the determination and selection of the next heading and step size, ensuring the formulation of an optimal, collision-free, and physically feasible trajectory between the start and target positions. This guidance facilitates the USV’s navigation, aiming to minimize path length, time, energy consumption, and other objectives [4,5,6]. Currently, mainstream path planning methods for USVs encompass traditional algorithms (e.g., Dijkstra and A*), intelligent algorithms (e.g., Genetic Algorithm (GA), Ant Colony Optimization (ACO), Particle Swarm Optimization (PSO), among others), and hybrid methods that integrate deep learning with reinforcement learning (such as Deep Q-Network (DQN), Deep Deterministic Policy Gradient (DDPG), Proximal Policy Optimization (PPO), Soft Actor–Critic (SAC), etc.) [7]. These methods diverge primarily in their approach to environmental perception. For traditional and intelligent algorithms, environmental perception primarily hinges on pre-constructed environmental models or the amalgamation of sensor information for environmental modeling. For instance, Zhang et al. [8] employed the sensor detection range to represent locally unknown dynamic environments and assessed environmental complexity through the virtual force field effect of an artificial potential field. Liu et al. [9], on the other hand, utilized clustering algorithms to perceive the environment, accurately determining the number of obstacle categories based on the aggregation degree of obstacles, thereby evaluating environmental complexity. However, the majority of these studies rely on the detection of a limited number of simple parameters to deduce the complexity of the surrounding environment, which fails to adequately capture the intricacies of real-world scenarios. Consequently, the accuracy in identifying environmental complexity is compromised. Issues such as excessive attractive force [10] and the challenge of parameter tuning can readily lead to errors in obstacle recognition when using the artificial potential field method, thereby limiting its practical applicability. Furthermore, the clustering algorithm struggles to identify clusters of different sizes and densities [11] and exhibits randomness in its iterative process [12]. This may potentially result in unstable clustering outcomes and an undue reliance on the quality of the input data.

With the continuous development of information technology, significant advancements have been made in computer vision and machine learning technologies, opening up new avenues for environmental perception. Consequently, many scholars have utilized deep reinforcement learning methods, leveraging deep learning models such as CNNs and RNNs, to extract environmental features for perception, classification, and recognition tasks. For instance, Zhao [13] proposed a specific environmental perception method for a power distribution room using a U-Net segmentation network combined with the ID information of electrical control cabinets. Jiang [14] introduced SHRDet, a lightweight sea surface target detection network designed for accurate and rapid detection of targets on the sea surface. Sorial et al. [15] employed CNNs to process fused data from camera and LiDAR sensors, achieving real-time obstacle detection and avoidance. Li et al. [16] proposed a method based on a multi-layer convolutional neural network to optimize the classification of synthetic aperture radar image data sources. Zhu et al. [17] addressed the issues of lack of optimization and computational resource waste in small target detection algorithms by proposing a lightweight small target detection algorithm based on multi-scale cross-layer feature fusion. Yang et al. [18] utilized a deep convolutional residual network to extract log-Mel spectrogram features of voiceprint information and employed the YOLOX-S network to extract optical features of targets, proposing a target recognition method that integrates acoustic and optical modalities. These studies underscore the potential and effectiveness of computer vision technologies in environmental perception tasks. By leveraging these models, researchers are able to accurately extract and identify environmental features, thus facilitating the construction of more intelligent and efficient systems. Consequently, the research motivation behind utilizing computer vision for environmental perception lies in leveraging the models’ powerful feature extraction capabilities to enhance the accuracy, efficiency, and robustness of environmental perception systems.

Given the aforementioned scenarios, to solve the issue of poor environmental perception without considering the current status of USVs in the optimization of heuristic algorithms, this paper proposes a lightweight CNN-based computer vision perception method that integrates current local environmental images and real-time heading angles for multi-feature fusion, then giving the right perceptional result about the local environment surrounding the relevant USV. Compared to existing lightweight models, including but not limited to MobileNet, ShuffleNet, and SqueezeNet, this lightweight CNN model maintains low total learnables and reduces model complexity while preserving recognition accuracy, which reduce the difficulty of its deployment. By comparing the environmental information captured by this model with labels of environmental complexity annotated based on human experience and incorporating real-time heading angles, the complexity of the current local environment for the USV can be determined. This environmental perception result is then fed back into the heuristic algorithm, thereby enhancing the optimization search performance.

## 2. Materials and Methods

### 2.1. Study Foundation

#### 2.1.1. Establishment of Environmental Model

As an underactuated marine vehicle with dynamic constraints, it is crucial to consider not only the practical conditions of a USV, such as spatial limitations, limited endurance, and minimum turning radius, but also to rapidly and accurately perceive and discern the complexity of its dynamic environment. To achieve this, environmental modeling must first be conducted, serving as the foundation for computer vision perception.

**Figure 1:**
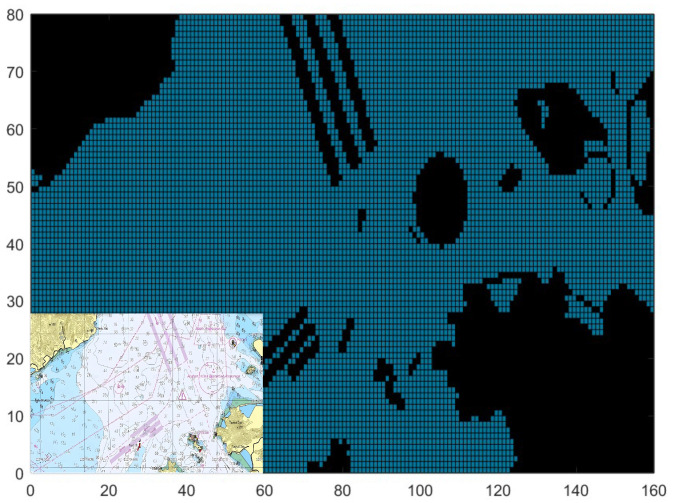
Grid map from electronic chart of designated area: black for obstacles, and azure for free navigable areas.

To guarantee that the information source contains detailed and precise marine environment data, the S-57 electronic nautical chart of a specified sea area (as depicted in the bottom-left corner of Figure 1) is adopted as the research backdrop. The marine environment information within the electronic nautical chart undergoes gray-scale conversion and binarization to establish a static grid-based marine simulation model, as shown in Figure 1. The gridded representation of the marine environment retains the land and island information from the original electronic nautical chart, treating sea highways and anchorages as static obstacles. Within this grid, black represents obstacles, while azure signifies freely navigable areas.

#### 2.1.2. Updating Dynamic Obstacle Environments

**Figure 2:**
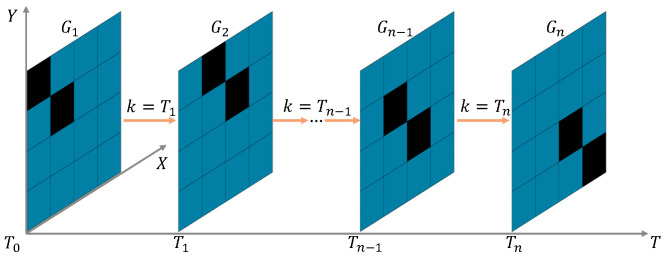
Static sequence samples of moving obstacle model.

Through the Automatic Identification System (AIS), information such as the size, speed, and heading of ships navigating at sea over a certain period can be obtained. The ships are modeled as part of a grid-based model using the method proposed in reference [19]. When converting AIS data into an obstacle model within the environment using the rasterization method, the process begins by screening and removing duplicate and erroneous data records through similarity comparisons, as well as synchronizing the timestamps of the data. Then, the latitude and longitude coordinates are converted into a Cartesian coordinate system. Subsequently, ships are simplified as point masses for grid occupancy marking. The rasterizing of obstacles is calculated by using the following formulas to determine their positional coordinate relationships at the center of each grid. Finally, an interval period is set to periodically read new AIS data and update the obstacle model. In this way, a set of position samples for dynamic obstacles at different time points is obtained on the grid map, forming multiple static sample sequences along the time axis, as shown in Figure 2.

(1) $$\begin{array}{cc} & X:x_{i}=a\times \left(\mathrm{mod}\left(i,MM\right)-0.5\right)\\ & Y:y_{i}=a\times \left(MM+0.5-\mathrm{ceil}\left(\frac{i}{MM}\right)\right) \end{array}$$

where *a* represents the side length of each grid, MM denotes the maximum grid value for both the horizontal (*X*) and vertical (*Y*) coordinates, the total number of grids is $MM^{2}$, $\left(x_{i},y_{i}\right)$ stands for the grid occupancy coordinates of the obstacle, *i* is the sequence index value for each grid, mod indicates the modulo operation, and ceil represents the ceiling function (rounding up to the nearest integer).

By updating these static sample sequences, the environmental model is transformed into a time-sharing dynamic model. The update mechanism for this transformation is defined as follows:

(2) $$G_{k}=\left\{\begin{array}{cc} G_{1}, & k=1,2,\ldots ,T_{0}\\ G_{2}, & k=T_{0}+1,T_{0}+2,\ldots ,T_{1}\\ \vdots & \vdots \\ G_{n}, & k=T_{n-1}+1,T_{n-1}+2,\ldots ,T_{n} \end{array}\right.$$

where $G_{1},G_{2},\ldots ,G_{n}$ are sample sequences according to the sample-updating period *T*, $G_{k}$ is the currently selected navigation map, and *k* is a real-time updating parameter. Initially, k=1, and it increases incrementally with each subsequent local path planning iteration. When *k* increases to $T_{1},T_{2},\ldots ,T_{n}$, $G_{k}$ is updated for the next local path planning session. $T_{1},T_{2},\ldots ,T_{n}$ are integer multiples of *T*, respectively, which can be selected based on dynamic real-time requirements. The smaller the multiple value, the higher the real-time performance.

This update mechanism is used to simulate complex dynamic marine environments and provides an environmental foundation for subsequent environmental perception.

### 2.2. Perception of Environmental Complexity in Computer Vision

In order to recognize and classify the complexity of the local environment surrounding the USV, computer vision methods are introduced with the aid of manually labeled experience-based classifications, aiming to reduce the error rate of environmental recognition.

#### 2.2.1. Lightweight Residual Learning Multi-Feature Fusion CNN

- Custom Lightweight CNN Architecture

**Figure 3:**
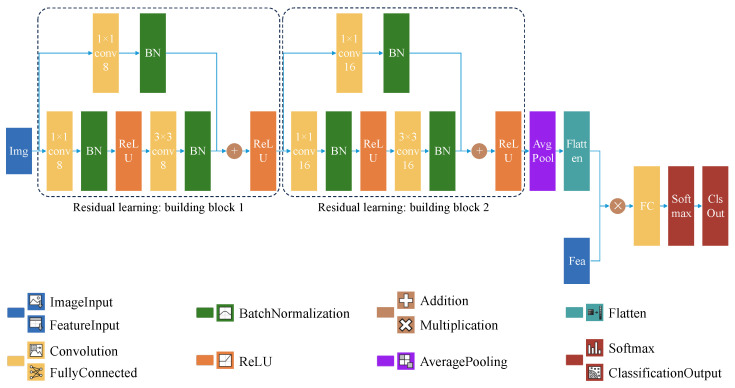
Custom lightweight CNN model.

Given the variability in complex perception of local environments, this paper proposes a multi-feature fusion model based on ResNet that employs residual learning. The advantage of residual learning lies in the addition of shortcut connections, which enable the model to neither add additional parameters nor increase computational complexity during the process of deep expansion, while simultaneously achieving higher accuracy gains [20]. Inherently, this model is compact, featuring a minimal number of parameters and ease of deployment, thereby making it ideal for local grid environments. The proposed lightweight model is illustrated in Figure 3.

As input to the model, during dataset preparation, each local environment image is saved with a unique filename corresponding to its defined heading angle and the complexity label determined by a relative majority voting system. During the training process, after loading a local environment image, the heading angle and label of each image are directly extracted from their respective filenames. This ensures a one-to-one correspondence between the heading angle features and the image features, allowing the current heading angle features to be fused with the corresponding feature maps to form a new composite feature space. This enhances the ability to discriminate between different environmental complexities corresponding to different heading angles in the same local environment.

Considering the actual pixel size of the images used, to avoid issues such as incomplete sliding of the convolution kernel over the image, which could hinder convolution operations and lead to dimension mismatch, up-dimensionality filters and feature extraction filters are configured. These filters have convolution kernel sizes of 1×1 and 3×3, respectively. And the corresponding output channel numbers are set to 8 and 16. The zero padding and stride for each convolutional layer are both set to 1, ensuring effective image convolution and extraction of key features.

After convolution, the feature tensors undergo batch normalization processing. Firstly, the cross-dimensional mean ($\mu_{B}$) and variance ($\sigma_{B}^{2}$) are calculated, and the input elements $x_{i}$ are normalized [21]. Subsequently, each normalized activation value is calculated as follows:

(3) $${\hat{x}}_{i}=\frac{x_{i}-\mu_{B}}{\sqrt{\sigma_{B}^{2}+\epsilon }}$$

Here, ϵ serves as a constant to enhance numerical stability, particularly when $\sigma_{B}^{2}$ is very small. When the zero-mean, unit-variance inputs resulting from batch normalization are not optimal, the following transformation is applied to further shift and scale the activation values:

(4) $$y_{i}=\gamma {\hat{x}}_{i}+\beta$$

Were the offset β and the scaling factor γ are learnable parameters that are updated during the model training process.

After batch normalization, the Rectified Linear Unit (ReLU) function is applied to activate each element of the input tensor, preventing issues of gradient vanishing or exploding, which in turn makes the model easier to train.

The image input processed by two residual blocks has its spatial dimensions folded into channels through a flattening operation. A multiplication layer fuses these data with the heading angle features input. The fully connected layer then classifies the fused features as either “complex” or “simple”, with its output matching the label categories in the dataset.

At this point, the basic architecture of the model has been completed. When constructing the CNN model, the specific layer architecture design and selection of key parameters are primarily based on ResNets [20]. Additionally, in order to enable the local environment complexity awareness and then provide feedback to the ACO algorithm for optimizing search, a lightweight model is developed by reducing the layer and channel, whilst the repeated trial-and-error and the comparative analysis are also conducted. The focus is on practical optimization rather than seeking in-depth theoretical analysis and derivation.

- Creation of Image Datasets

**Figure 4:**
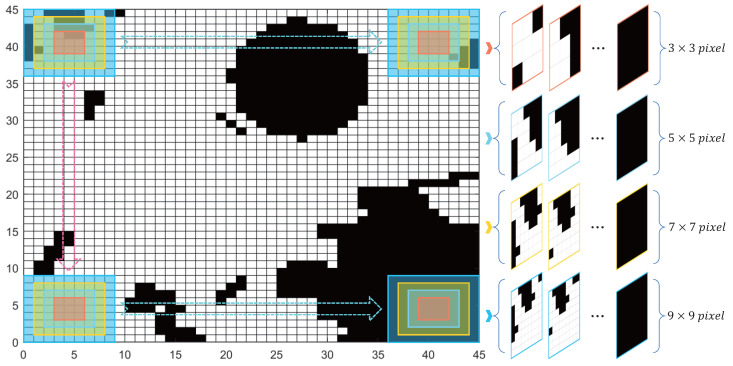
Segmentation and extraction of local environmental grid maps with different search step sizes.

Due to the specific nature of identifying and classifying the complexity of the local environment, it is not feasible to directly use public datasets such as MNIST, CIFAR-10, and ImageNet, along with related pre-trained models (e.g., GoogLeNet, ResNets, Inception). Instead, the marine grid model is segmented into various pixel sizes (as depicted in Figure 4) to extract local environmental images that correspond to various search step sizes. These images are then used as the training and testing sets for the CNN. It is noteworthy that, due to the need for multi-input feature fusion with the heading angle of the USV, no image enhancement processing is applied to the segmented image dataset obtained in this work; through this, the potential interference that may lead to misdirection during the perception can be avoided.

**Figure 5:**
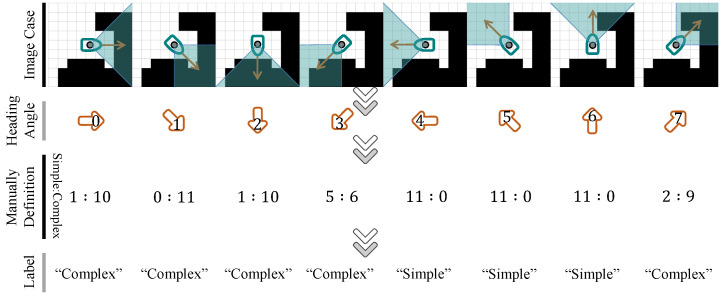
Image examples of the decision procedure for complexity labels.

Centering on the current location of the USV, four sets of image datasets are obtained with step sizes corresponding to radii of 1–4 steps (3×3, 5×5, 7×7, and 9×9 pixels). These sets contain 2118, 3590, 4664, and 5262 samples, respectively. Each set of samples is manually classified into “complex” and “simple” categories based on eight different heading angle empirical classification labels. Figure 5 showcases a set of image examples from the process of assessing complexity labels. During the specific implementation, the distribution of obstacles (represented as black grids) within the $\pm 45^{\circ }$ visual range corresponding to the USV’s heading angle in the local environment, as well as the feasibility of reaching the local environment boundary directly along the current heading angle, are taken into account. Eleven individuals independently vote based on a relative majority voting system, and the aggregated results determine the complexity label. Subsequently, for each complexity category, the samples are randomly divided into training and testing sets in an approximate 8:2 ratio to facilitate the training process.

- Definition of Heading Angle Characteristics

**Figure 6:**
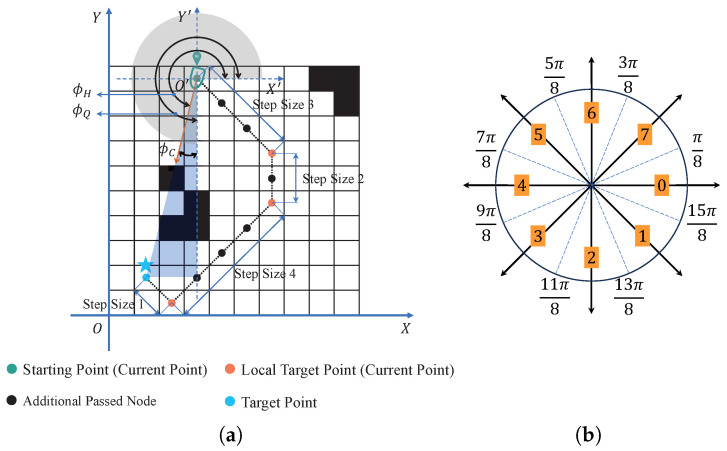
2π circumferential heading angle feature extraction: (**a**) Course angle determination. (**b**) Course angle feature.

For angular feature input, the initial heading angle is determined directly by the cosine relationship between the starting point and the target point in the grid map. Subsequent angles are also determined in a similar manner during the iterative process, but now between the current point and the local target point, as illustrated in Figure 6a. To standardize the input, angles within the 0–2π range are divided into eight counterclockwise intervals, starting from 0 degrees, and represented by integers 0–7 (as shown in Figure 6b).

Here, $\phi_{H}$ denotes the heading angle, $\phi_{Q}$ represents the quadrant angle, and $\phi_{C}$ signifies the cosine angle formed between the current point and the local target point. The relationship among them is expressed as $\phi_{H}=\phi_{Q}-\phi_{C}$. In the $X^{\prime }O^{\prime }Y^{\prime }$ coordinate system, which has the current point as the origin and is parallel to the XOY coodinate system, the heading angle $\phi_{H}$ equals $\phi_{C}$ when the USV is directed in the first quadrant. When the USV is directed in the second, third, or fourth quadrant, $\phi_{Q}$ takes the values of π, $\frac{3\pi }{2}$, and 2π, respectively.

#### 2.2.2. Model Training, Testing, and Evaluation

The customized lightweight model architecture encompasses a total of 26 layers. In comparison to the MobileNet, ShuffleNet, and SqueezeNet series [22], its total number of learnable parameters is merely 223.2K (see Table 1). Disregarding the additional floating-point operations (FLOPs) stemming from bias terms, the model boasts just 163.37M FLOPs. As shown in Table 1, when receiving the same input tensors and with an accuracy loss not exceeding 1.5%, the constructed CNN achieves a 61% greater simplification in its architectural hierarchy, reduces the total number of learnables by over 69%, and lowers the model complexity by approximately 46%. This demonstrates significant advantages compared to some existing lightweight networks. Although the metrics considered here are not definitive standards for evaluating the superiority of a network, they do emphasize the lightweight characteristics of the constructed network. Furthermore, this characteristic has a substantial impact on the practical deployment of CNNs for environmental perception in USVs. Consequently, we conclude that the model possesses lightweight characteristics. While maintaining a certain depth and width, the model keeps the number of learnable parameters low, thereby enhancing its feature extraction capability for image recognition and simplifying the processes of model training and testing. Below are the specified model training options for four distinct step sizes. The optimizer utilized is SGDM, with a batch size of 32 and MaxEpochs set to 1000. The initial learning rate begins at 0.01, accompanied by a weight decay of 0.01 and a momentum of 0.9. During the training process, the learning rate is reduced once every 100 epochs with a decay factor of $10^{-2}$, aimed at lowering the global learning rate. Data shuffling precedes each training epoch. An L2 regularization factor of 0.0001 is chosen, and a threshold method is applied to scale the gradients of the learnable parameters. The construction and training of the model are conducted using a deep learning toolbox on a single NVIDIA RTX 4060 GPU with 8GB of memory, terminating upon reaching a predefined MaxEpoch value.

The trained model is evaluated using a confusion matrix on the test set. Taking the CNN-SS1 model as an example, 424 images were tested, with 358 “simple” images and 23 out of 59 “complex” images being correctly classified, whereas 36 images were misclassified as “simple”. The confusion matrix allows us to assess the model’s accuracy (Acc), precision (Pre), recall (Rec), and F1 score (F1) [23]. Accuracy represents the probability of correct classification, precision indicates the proportion of positive samples among the identified images, recall signifies the proportion of correctly identified positive samples, and the F1 score is a metric that harmonizes both precision and recall [24].

The Acc, Pre, Rec, and F1 values of the model are presented in Table 2. The results indicate that, for datasets manually annotated with different step sizes, this customized lightweight model achieves recognition accuracies of 89.86%, 89.84%, 90.74%, and 89.36% on four respective datasets. The constructed residual learning multi-feature fusion model fulfills the requirements for recognizing the complexity of the local environment.

#### 2.2.3. Ablation Experiment

**Figure 7:**
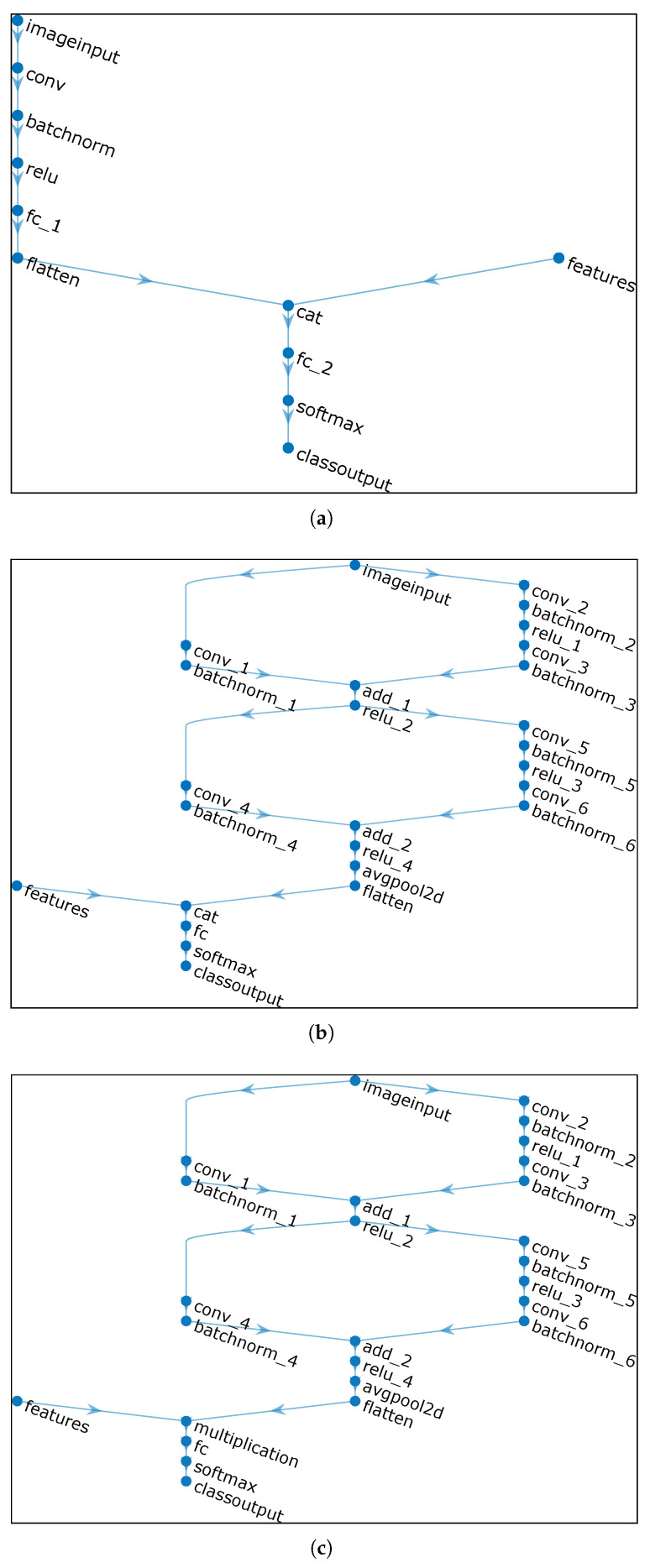
Simplified architecture of model used in ablation experiments: (**a**) Model architecture-1. (**b**) Model architecture-2. (**c**) Model architecture-3.

To validate the effectiveness of the custom lightweight residual learning multi-feature fusion model, this paper designs ablation experiments, primarily assessing the differences in performance efficiency of the model by removing or modifying network structure components. The image datasets and heading angle features utilized in the ablation experiments remain consistent, and identical training parameter settings are employed during both the training and testing phases. The only comparison conducted is how different model architecture designs affect environmental complexity perception performance. Figure 7 illustrates the simplified architectures of the models employed in the ablation experiments. Figure 7a presents a baseline, providing a general framework for the multi-input architecture model. Figure 7b,c incorporate residual learning blocks into this baseline. Model architecture-1 and -2 connect the fully connected layer through an addition layer, whereas model architecture-3 connects the fully connected layer via a multiplication layer. Modifications and tests are conducted on the models in this manner to achieve the purpose of ablation.

**Figure 8:**
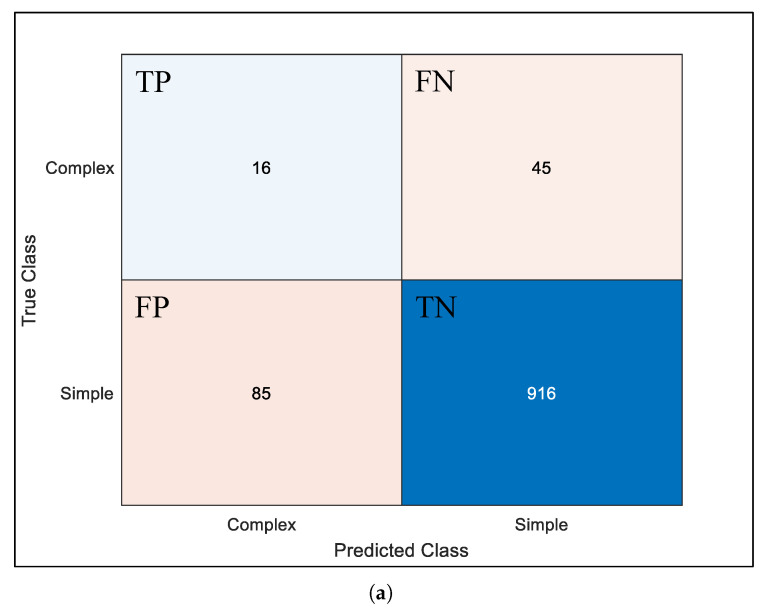

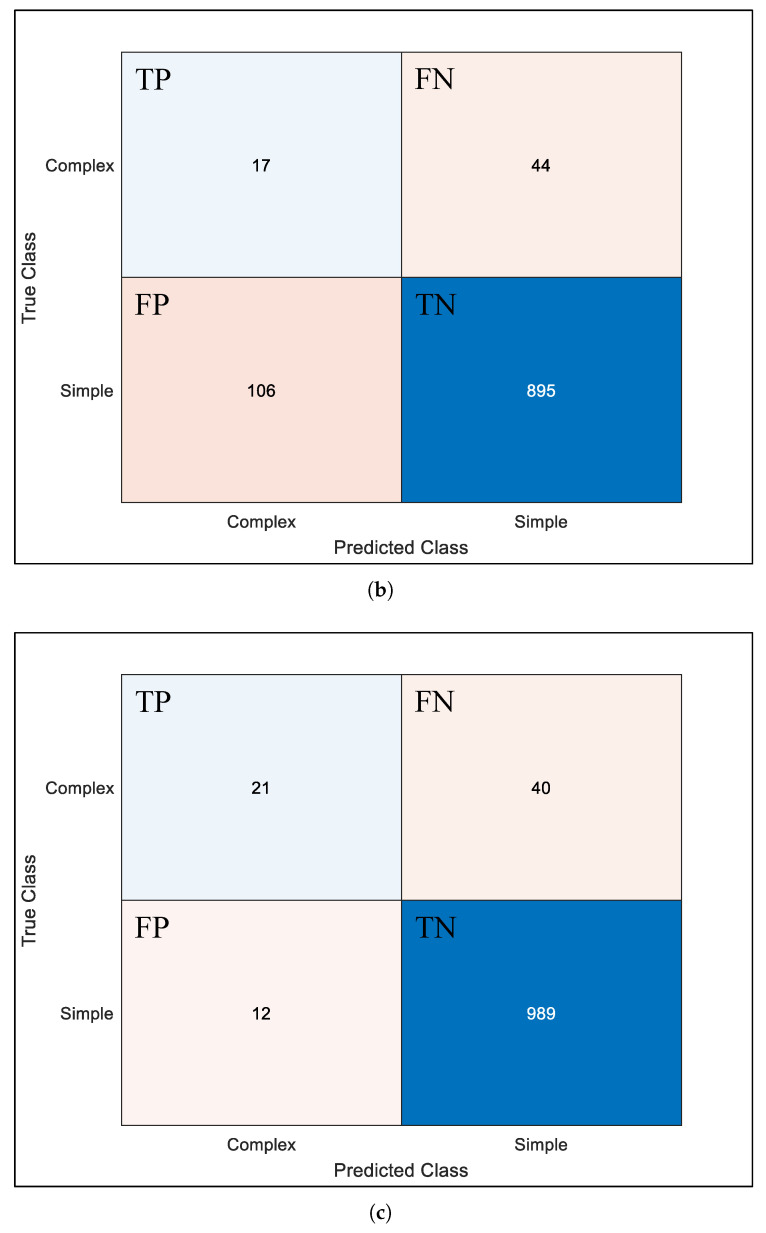
Comparative analysis of model confusion matrix in ablation experiments: (**a**) Confusion matrix of architecture-1. (**b**) Confusion matrix of architecture-2. (**c**) Confusion matrix of architecture-3.

Using the same dataset, short-round training and testing sessions were conducted on models featuring different architectures, and the confusion matrices obtained for each are depicted in Figure 8. The confusion matrices in Figure 8a–c correspond to model architecture-1, -2, and -3, respectively, in Figure 7. The ordinate represents the real category, that is, the existing labels in the testing set. And the abscissa denotes the prediction category, namely, the model’s prediction of the testing set. Here, TP, TN, FP, and FN are true positive, true negative, false positive, and false negative recognition, respectively. From Figure 8, it can be observed that there are slight differences in the environmental perception results among the various architectures. In terms of accuracy, architecture-3 scores 95.10%, surpassing the 85.88% of architecture-2 and the 87.76% of architecture-1. When considering the recall rate, architecture-3 (34.43%) outperforms the other two by nearly 6.56 percentage points over architecture-2 (27.87%) and 8.07 percentage points over architecture-1 (26.23%). This demonstrates that architecture-3 is more proficient in recognizing and classifying “complex” environments, which is crucial for environmental perception. Moreover, one can find that the accuracy, precision, and recall metrics of the lightweight CNN have been optimized in terms of the ablation approach. However, it is evident from the confusion matrix that the model’s precision and recall metrics remain relatively low. This fact reflects the occurrence of misclassification during the model testing process. This phenomenon can be attributed to the skewed sample distribution within the dataset, which unexpectedly mirrors the structural parameters of the developed marine environment model. Additionally, a certain degree of ambiguity in manual empirical labeling needs to be accommodated. In conclusion, architecture-3 is chosen as the final architecture for the customized lightweight residual learning multi-feature fusion model.

After the customization, training, testing, and ablation experiments of the lightweight CNN architecture, the network model was successfully constructed. Once the CNN architecture was finalized, it was utilized within the constructed marine environment model to recognize and classify the current local environment as either “complex” or “simple”, based on the perception area of the USV’s location. Upon obtaining the CNN’s perception results, these results were fed back into the ACO algorithm for a more detailed determination of complexity classification levels, and different step sizes were selected accordingly based on these complexity classification levels. Subsequently, the next perception point was chosen based on the selected step size. This perception feedback-to-step size decision matching strategy is implemented through Algorithm 1. The algorithm receives preliminary perception feedback from the CNN model concerning the local environment and performs a more granular classification of complexity levels for different perception areas by using a progressive comparison with a composite ladder approach, employing an “AND” logic gate. This results in complexity levels spanning from 1 to 4, where Level 1 signifies the most complex situation and the complexity diminishes as the level ascends. Ultimately, the decision on step size is made based on this detailed complexity classification level. Utilizing this perception feedback and step size decision strategy, further testing and validation of the proposed visual perception method can be undertaken through numerical simulations.
**Algorithm 1** CNN complexity recognition and level evaluation matching strategy.**Input:** 
$W_{c}$, $A_{s}$, *G*, MM
**Output:** 
Complexity, RoSS
  1: $Complexity\left(i\right)\leftarrow CNN\_SS_{i}\left(W_{c},A_{s},G,MM\right),i=1,2,3,4$
  2: **if** Complexity(1)= “Complex” **then**
  3:    RoSS=1
  4: **else** **if** Complexity(1)= “Simple” **then**
  5:     **if** Complexity(2)= “Complex” **then**
  6:        RoSS=1
  7:      **else if** Complexity(2)= “Simple” **then**
  8:         RoSS=2
  9:         **if** Complexity(3)= “Complex” **then**
10:              RoSS=2
11:         **else** **if** Complexity(3)= “Simple” **then**
12:              RoSS=3
13:
             **if** Complexity(4)= “Complex” **then**
14:                   RoSS=3
15:              **else** **if**  Complexity(4)= “Simple” **then**
16:                   RoSS=4
17:              **end if**
18:          **end if**
19:        **end if**
20:   **end if**


## 3. Methods Validation and Analysis

### 3.1. Configuration of Simulation

Taking into account the direct requirements of environmental recognition tasks for obstacle judgment, as well as the comprehensive considerations of computational efficiency, real-time performance, and scenario needs, this study did not excessively pursue comprehensiveness and diversity in simulation comparisons. Instead, we adopted two different methods: computer vision perception and clustering algorithm perception. CNN perception employs the convolution operation of convolutional kernels to extract features from environmental image data, and incorporates manually labeled experience to perform complex classification. The K-Means clustering algorithm processes environmental information by iteratively assigning obstacle data based on Euclidean distance to find the optimal cluster partition in the absence of cross-validation. The main object is to explore the differences in effectiveness between two classes of approaches when employing different perception methods to sense the environment and feeding this information back into the ACO algorithm for exhaustive optimization search. Thus, comparative simulations of environmental complexity perception are conducted using these two methods in a 45 × 45 grid model environment.

Based on the discussion in Section 2, the primary factors influencing model performance are data, model architecture, training strategies, and dynamic environmental disturbances. The size variation of the grid map does not significantly impact the environmental perception performance of the CNN model. This is because the model’s perception area focuses on the local environment, rather than perceiving the entire grid map directly. Furthermore, not all points in the grid are treated as perception points for local environmental perception. Only the points selected through ACO search within the perception area are designated as perception points. And the first three factors affecting model performance have been thoroughly investigated in the previous sections. Thus, in this simulation section, the recognition and generalization capabilities of the proposed environmental perception method were tested by periodically updating the environmental model to introduce dynamic environmental disturbances. All simulations were conducted under identical conditions to ensure consistency in testing (i.e., Windows11 64-bit operating system, Intel® Core™ i9-13900H 2.60 GHz processor, 16 GB memory laptop). Then, the environmental perception results from the two methods are fed back into the ACO heuristic algorithm. The basic parameter value settings for each method are shown in Table 3.

### 3.2. Perception of Local Static Environment

**Figure 9:**
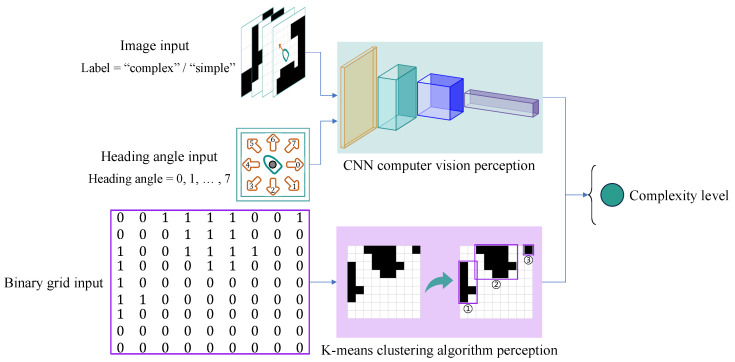
CNN-based (green-boxed) vs. K-Means-based (purple-boxed) perception.

To assess the complexity of the local static environment, a limited number of environmental perception tests were conducted within a 9×9 pixel local environment characterized by a low density of obstacle distribution. For the computer vision perception method, various local environment images and heading angle features were utilized as inputs for the constructed model. For the clustering algorithm perception, the input required was the binarized values corresponding to the local environment images (where “0” signifies passability and “1” signifies an obstacle), as illustrated in Figure 9.

**Figure 10:**
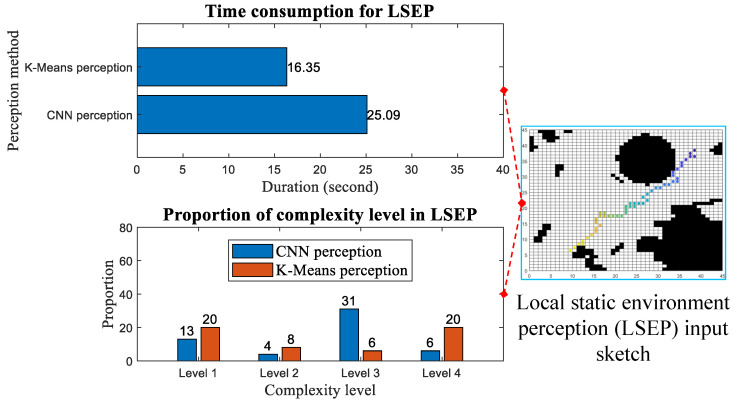
Comparison of local static environmental perception (The colored dots on the grid map represent the perception and search points traversed from the initial to the designated perception point.).

**Figure 11:**
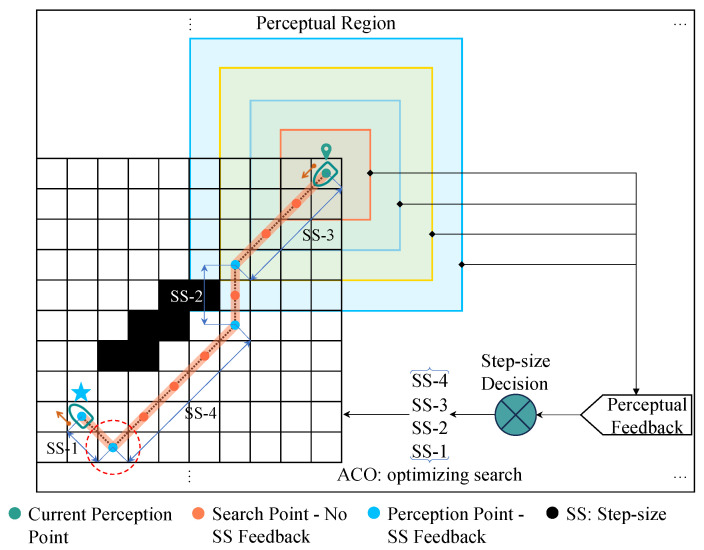
Illustration of environmental perception and feedback for step size decision-making.

Figure 10 depicts the proportion of perceived environmental complexity levels following the tests conducted using the two distinct perception methods. When conducting local static environment perception, only a single grid map is utilized to provide environmental information, as shown on the right side of Figure 10. During this process, all obstacles remain static, without dynamic updates being performed. The points in the grid map visualize the positions of the optimized search results from point to point by the ant colony in the ACO algorithm based on environmental perception feedback. Figure 11 demonstrates the specific process where the USV, after perceiving the environment at a perception point, makes decisions with varying step sizes and feeds these decisions back to the ACO algorithm to select the next perception point corresponding to the step size. On the way from the current perception point to the next, ants pass through a varying number of search points within the perceptual area (equivalent to this area in terms of step size) to conduct optimizing searches step and determine the next perception point until they reach the designated point. During this process, environmental complexity perception and feedback are only conducted at the selected perception points and no perception or step size feedback occurs at the search points. The left side of Figure 10 presents statistical results for point-to-point local static environment perception using two different perception methods. The comparison of results reveals that, although adopting CNN perception sacrifices a certain time advantage (the duration indicated in the figure refers to the time consumed for completing the point-to-point environmental perception and step size decision iteration, starting from the initial perception point to the designated perception point, and this duration is obtained using a computer timing program), it achieves more accurate environmental complexity results compared to K-Means perception. The proportion of Level 1, which indicates high complexity levels, remains at a low level, consistent with the trend in the dataset sample proportion.

Table 4 documents the differences between CNN perception and K-Means perception in the same local static environment, with the inclusion of heading angle features, highlighting that visual perception, when combined with heading angle in the same environmental map, can achieve more targeted complexity recognition results. It is evident from the table that the environmental complexity features obtained by these two perception methods at the same perception points in the same environment exhibit significant differences. Across the three sets of perception points, CNN perception consistently extracts more targeted environmental information as the real-time heading angle changes, resulting in varying complexity scenarios, which is highly beneficial for USVs. However, K-Means perception lacks the ability to integrate heading angle features, leading to a singular complexity scenario regardless of the heading angle within the same environment. This advantage arises from the feature fusion capability of the multi-input lightweight model architecture, enabling the CNN perception method to capture differentiated environmental complexity features across different heading angles. This capability is crucial for facilitating efficient optimization searches by ant colonies towards specified target points, thereby ensuring a more rational and effective selection of the next perception point. Consequently, the increased time consumption associated with using the CNN visual method for environmental perception is greatly compensated for during the optimization search process of ACO, and this compensation effect is superior to that achieved with K-Means perception.

### 3.3. Perception of Global Dynamic Environment

**Figure 12:**
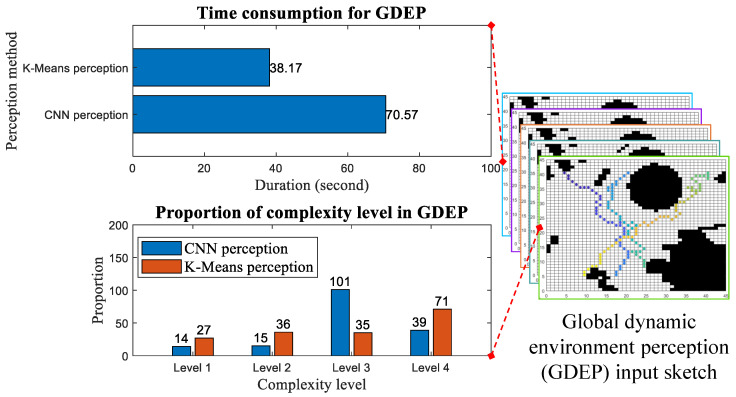
Comparison of global dynamic environmental perception (The colored dots on the grid maps represent the perception and search points traversed from the initial to the designated perception point.).

To further validate the proposed method, a comparative simulation of complexity perception in a global dynamic environment is conducted for two sensing approaches. Unlike local static environment perception, global dynamic environment perception requires dynamically updating multiple grid maps obtained from the AIS, which contain information about various moving obstacles, using the method described in [19]. This approach enhances the random dynamics of obstacles, thereby increasing the difficulty for the proposed sensing method to extract features from environmental information at different sensing points when confronted with unknown dynamic environments. The proportion of environmental complexity levels perceived by different methods is shown in Figure 12. The right side of Figure 12 also visualizes the continuous environmental sensing and feedback-optimized search processes during global dynamic environment perception. The left side of Figure 12 presents statistics on the results of the two methods after performing point-to-point global dynamic environment perception. It is evident that the time consumed by both methods for global dynamic environment perception increases significantly compared to local static environment perception due to the dynamic updates (this duration is also obtained using a computer timing program). However, the CNN-based sensing method is still able to achieve a relatively low proportion of high complexity (Level 1), indicating that the constructed lightweight CNN model possesses certain transfer and generalization capabilities in simulation applications.

Through the aforementioned simulations, the effectiveness of CNN-based visual perception can be verified. Additionally, for further validation, we integrated visual perception and clustering perception with the adaptive ant colony algorithm proposed in [9] to conduct simulation tests for dynamic path planning and evaluate the planning results. The purpose of this integration is to compare the enhancement in optimization search performance that the visual perception feedback method could provide to heuristic algorithms. We selected three sets of simulations with identical start-to-goal points for 30 path planning runs. Various evaluation metrics were defined, and the average metrics for each set of simulations were calculated. The results of these simulations are recorded in Table 5.

Among the three sets of simulations (“⊝”, “⊚”, and “⊛” representing different sets with the same start-to-goal points), dynamic path planning using visual perception achieved varying degrees of optimization in terms of the number of traversed nodes (NTN, in unit), turning angle (TA, in radians), time consumption (TC, in seconds), and planning success rate (APR, in percentage). Compared to planning with clustering perception, CNN-based perception planning reduced NTN by 1.96% to 25.00%, TA by 2.19% to 55.30%, and TC by up to 71.30%. Additionally, it increased APR by 6.67% to 30.00%. CNN-based perception planning also reduced the number of turns (TT, in occurrences) in all sets. In terms of path length (PL), CNN-based perception planning resulted in shorter paths than clustering perception planning in all sets, with a more pronounced advantage when planning longer paths. To this end, the proposed visual perception method for assessing local environmental complexity in USVs demonstrated superior performance compared to clustering perception in terms of NTN, TT, TA, TC, PL, and APR, and the overall performance of the optimized search planning was improved by approximately 21%.

Based on the aforementioned various aspects, simulations and analysis were conducted to verify the effectiveness of the proposed method.

## 4. Conclusions

The conventional ACO algorithm exhibits limitations in sensing environmental factors, especially in environments laden with intricate and unpredictable dynamic obstacles, inevitably resulting in misidentification issues. To overcome this issue, this paper refrains from solely depending on iterative computation of obstacle data for acquiring and assessing environmental conditions. Instead, it incorporates artificial experience-derived classification labels to devise a streamlined residual learning multi-feature fusion CNN model for assessing local environmental complexity. Simulations grounded in local static environmental awareness and global dynamic environmental perception reveal that this model exhibits superior intelligent traits in sensing environmental complexity. Without unduly prioritizing model precision, it can still yield perception outcomes of environmental complexity across various heading angles, which is advantageous for path planning issues for USVs.

This paper aims to enhance the accuracy of identifying environmental complexity during search planning for USVs by improving the capability to fully extract and perceive environmental features, through the integration of computer vision methods with heuristic optimization algorithms. The feasibility of this method has been validated through simulation experiments, and preliminary tests have been conducted using the ROS robotic system, where the range data obtained from the onboard ToF LiDAR sensor are utilized for real-time mapping and perception with the constructed CNN. However, in actual environmental perception tasks, USVs encounter challenges arising from sensor performance and other comprehensive environmental factors, necessitating a higher level of reliability and effectiveness for this perception method. Consequently, future endeavors will concentrate on conducting pertinent real-ship trials in actual application scenarios.

## Figures and Tables

**Table 1:** Comparison of lightweight models.

Models	Input Resolution	Evaluation Indicators			
		Layers	Total Learnables	FLOPs (M)	Acc (%)
Proposed	** 224×224 **	26	223.2K	163.37	89.36
SqueezeNet		68	722.3K	35,019.96	90.07
MobileNetV2		154	2.2M	306.18	90.67
ShuffleNet		176	862.3K	300.72	90.56

**Table 2:** Performance evaluation index of local environmental complexity identification and classification models.

Models	Evaluation Indicators			
	Pre (%)	Rec (%)	F1	Acc (%)
CNN_SS1	76.67	38.98	51.68	89.86
CNN_SS2	53.57	38.46	44.77	89.84
CNN_SS3	32.00	23.19	26.89	90.74
CNN_SS4	22.92	36.07	28.03	89.36

**Table 3:** Basic parameter value setting of each method.

Parameters	Initial Value	
	CNN	K-Means
Pheromone factor	1	1
Heuristic factor	14	14
Pheromone volatilization coefficient	0.3	0.3
Pheromone concentration enhancement factor	1	1
Number of ants	12	12
Maximum iterations	10	10
Number of relay points	2	2
Step size	1–4	1–4
Number of clusters (k)	-	4

**Table 4:** Difference in perception with or without heading angle.

Perception Point	Perception Methods	Complexity Levels of VHA							
		0	1	2	3	4	5	6	7
366	CNN	2	3	3	3	4	4	4	4
	K-Means	2							
1556	CNN	4	4	4	4	4	4	1	1
	K-Means	3							
1445	CNN	1	1	3	4	4	4	4	4
	K-Means	4							

VHA means “various heading angles”.

**Table 5:** Simulation results of planning performance for different perception methods.

Perception Methods	Evaluation Metrics					
	NTN	TT	TA	TC	PL	APR
CNN ^⊝^	77	41	35.3	32.3	58.4	96.6
K-Means ^⊝^	90	46	36.1	48.2	66.0	73.3
CNN ^⊚^	63	26	21.9	38.3	52.5	96.6
K-Means ^⊚^	73	31	24.3	45.4	58.4	90.0
CNN ^⊛^	50	21	18.1	30.3	41.5	96.6
K-Means ^⊛^	51	19	21.2	31.5	42.1	86.6

“⊝”: groups 1 with starting and target point coordinates at (41.5, 41.5). “⊚”: groups 2 with starting and target point coordinates at (3.5, 41.5). “⊛”: groups 3 with starting and target point coordinates at (6.5, 41.5).

## Data Availability

The original contributions presented in the study are included in the article; further inquiries can be directed to the corresponding author.
